# Interferon regulatory factor 4–releasing 3D-printed scaffolds enhance spinal cord repair by modulating macrophage polarization

**DOI:** 10.4103/NRR.NRR-D-25-00067

**Published:** 2025-09-29

**Authors:** Jianhao Wang, Jiawei Du, Tuo Fang, Di Zhang, Yigang Lv, Zhongju Shi, Hengxing Zhou, Shiqing Feng

**Affiliations:** 1 Department of Orthopedics, Tianjin Medical University General Hospital, International Science and Technology Cooperation Base of Spinal Cord Injury, Tianjin Key Laboratory of Spine and Spinal Cord, Tianjin, China; 2 Department of Spine Surgery, Peking University People’s Hospital, Beijing, China; 3 Department of Orthopedics, Qilu Hospital of Shandong University, Cheeloo College of Medicine, Shandong University, Jinan, Shandong Province, China; 4 Department of Orthopedics, the Second Hospital of Shandong University, Cheeloo College of Medicine, Shandong University, Jinan, Shandong Province, China; 5 Shandong University Centre for Orthopaedics, Advanced Medical Research Institute, Cheeloo College of Medicine, Shandong University, Jinan, Shandong Province, China

**Keywords:** 3D-printed scaffold, inflammatory microenvironment, interferon regulatory factor 4, JAK1/STAT6 signaling pathway, macrophage polarization, microglia, nerve regeneration, spinal cord injury

## Abstract

Three-dimensional (3D)-printed hydrogel scaffolds are widely used in spinal cord injury repair, with gelatin methacrylate being particularly favored owing to its excellent biocompatibility. However, traditional scaffolds have a small contact area with tissues and lack the ability to regulate the inflammatory microenvironment. Therefore, there is a need to develop smart scaffolds with drug delivery and immune regulation functions. In this study, a 3D-printed gelatin methacrylate scaffold was developed to deliver interferon regulatory factor 4 in a targeted and sustained manner. The scaffold showed good mechanical properties, biocompatibility, and sustained interferon regulatory factor 4 release. The sustained-release interferon regulatory factor 4 competitively bound to myeloid differentiation factor 88 to inhibit the pro-inflammatory effects of interferon regulatory factor 5, and activated the signal transducer and activator of transcription 6 pathway to promote M2 macrophage polarization, thereby facilitating neural regeneration and recovery of spinal cord function. This indicates that the constructed interferon regulatory factor 4-loaded 3D-printed methyl acrylate-modified gelatin scaffold can regulate macrophage polarization through the interferon regulatory factor 4/5 axis, improve the inflammatory microenvironment after spinal cord injury, and thus provide a new target for promoting neural regeneration.

## Graphical abstract

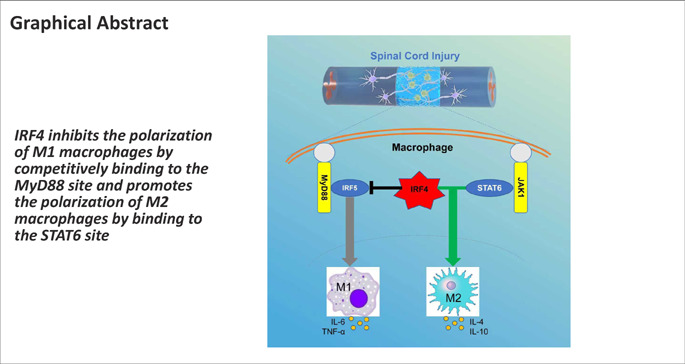

## Introduction

Spinal cord injury (SCI) is a major cause of long-term disability and mortality, particularly because of its limited capacity for self-repair. The initial mechanical trauma of SCI causes immediate damage, including destruction of neural tissue, disruption of axons, and vascular injury leading to hemorrhage (Anjum et al., 2020). Following the primary trauma, SCI initiates a complex cascade of secondary injury processes that lead to progressive tissue damage and functional deficits. These include inflammation (Hellenbrand et al., 2021), apoptosis (He et al., 2024), and oxidative stress (Visavadiya et al., 2016), all of which exacerbate the damage and extend tissue loss beyond the initial impact zone (Fan et al., 2022). Inflammation plays an important role in this process (DiSabato et al., 2024). Immediately after SCI, resident microglia and astrocytes become activated, releasing pro-inflammatory cytokines, such as tumor necrosis factor-alpha (TNF-α), interleukin-1β (IL)-1β, and IL-6 (Wang et al., 2024). This leads to the recruitment of peripheral immune cells, including neutrophils and macrophages, to the injury site (Li et al., 2022). Macrophages subsequently arrive, exhibiting both pro-inflammatory and anti-inflammatory roles, which are critical in determining the extent of secondary injury and repair processes (Fu et al., 2022; Xia et al., 2022). As the injury progresses, chronic inflammation can lead to the formation of a glial scar. Although the glial scar serves to contain the injury and prevent further spread of damage, it also poses a barrier to axonal regeneration, thereby impeding functional recovery (Pang et al., 2021; He et al., 2022).

Macrophages play a pivotal role in the inflammatory response following SCI, with their polarization into M1 and M2 phenotypes significantly influencing injury outcomes (Wang et al., 2023). Macrophage polarization into M1 and M2 phenotypes is critically regulated by several signaling pathways, which are activated in response to injury and environmental cues. The pathways related to reprogramming of macrophages include nuclear factor kappa-B (NF-κB), Janus kinase/signal transducer and activator of transcription (JAK/STAT), Toll-like receptor (TLR), phosphoinositide 3-kinase/protein kinase B (PI3K/AKT), and others (Yunna et al., 2020; Li et al., 2023; Toledo et al., 2024). This activates downstream pathways, including the MyD88-dependent pathway, leading to the activation of NF-κB and IRF5, which promote the inflammatory response and M1 macrophage polarization (Lv et al., 2022). Additionally, IFN-γ binding to its receptor activates JAK/STAT1 and PI3K/AKT pathways, further supporting M1 polarization (Yeh et al., 2015).

Interferon regulatory factor 4 (IRF4) is a key regulator of macrophage polarization, playing a crucial role in modulating the inflammatory response after SCI (Fu et al., 2022). As an important transcription factor in immune regulation, IRF4 facilitates M2 macrophage polarization by competitively binding to MyD88 (Qin et al., 2024). This shift is critical for resolving inflammation and enhancing tissue repair in SCI. Activation of IRF4 by signaling molecules such as IL-4 and IL-13 induces M2 polarization through signal transducer and activator of transcription 6 (STAT6) activation, thereby limiting excessive inflammation and supporting recovery (Tugal et al., 2013; Wang et al., 2023). The absence or dysregulation of IRF4 leads to impaired M2 polarization, as evidenced by reduced expression of M2 marker genes such as *Arg1*, *Ym1*, and *Fizz1*. This disruption in macrophage polarization contributes to increased tissue damage and hinders recovery (Satoh et al., 2010). Therefore, activating IRF4 represents a promising therapeutic strategy for reducing inflammation and reprogramming the injury site to support healing and tissue regeneration following SCI.

Recent advances in tissue engineering have led to the development of 3D-printed gelatin methacrylate (GelMA) scaffolds for SCI repair (Jiu et al., 2024). These scaffolds provide structural support and promote neural regeneration at the injury site (Koffler et al., 2019; Walsh et al., 2022). GelMA scaffolds have demonstrated excellent biocompatibility, with minimal cytotoxicity and favorable cellular interactions (Gao et al., 2023; Walsh et al., 2023). Implantation of the scaffold in rat models resulted in notable neural circuit reconstruction, reduced local neuroinflammation, and significant recovery of hindlimb motor functions (Qiu et al., 2023). Therefore, in the present study, we aimed to determine whether a 3D-printed GelMA scaffold loaded with IRF4 can modulate macrophage polarization toward an anti-inflammatory (M2) phenotype and thereby enhance SCI repair. We hypothesize that IRF4 exerts its effect by disrupting the IRF5–MyD88 interaction and promoting STAT6-mediated M2 polarization, ultimately improving the inflammatory microenvironment and functional recovery following SCI.

## Methods

### Molecular docking

Protein structures were obtained from UniProt (https://www.uniprot.org/). The complete structures predicted by AlphaFold were chosen for subsequent analysis. HDOCK software (https://hdock.phys.hust.edu.cn/) was used to perform flexible ligand docking simulations with a rigid protein and a flexible small molecule. Finally, PyMOL software (https://pymol.org/) was used to visualize the molecular docking results.

### Molecular dynamics simulations

Molecular dynamics simulations were conducted following an initial molecular docking procedure using the HDOCK program. The resulting docked complexes, including IRF5-MyD88, IRF4-MyD88, IRF4-MyD88-IRF5, and IRF4-STAT6, were subsequently subjected to a 50 ns molecular dynamics simulation in an aqueous environment. The system was modeled using the Amber99SB force field, with simulations carried out under standard conditions (25°C and 1 atm) to allow for system optimization. After performing energy minimization, the system underwent a 100 ps NVT equilibration followed by a 100 ps NPT equilibration. The final simulation was conducted for 50 ns following the pre-equilibration phase.

System 1: The docked IRF4–MyD88 complex was subjected to a 50 ns molecular dynamics simulation in aqueous solution. The system was constructed using Gromacs2021 (https://www.gromacs.org/) and consisted of 1 IRF4 protein molecule, 1 MyD88 protein molecule, and 103,202 water molecules, with 7 Na^+^ ions added to balance the charge in the system. The Amber99SB force field was used. The simulation box was set to a size of 11 nm × 12 nm × 12 nm and was run at 25°C and 1 atm. The simulation was optimized and run through a series of steps: energy minimization, 100 ps NVT, 100 ps NPT, and a 50 ns production simulation.

System 2: The docked IRF5–MyD88 complex was subjected to a 50 ns molecular dynamics simulation in aqueous solution. The system was constructed using Gromacs2021 and consisted of 1 IRF5 protein molecule, 1 MyD88 protein molecule, and 82,824 water molecules, with 12 Na^+^ ions added to balance the charge in the system. The Amber99SB force field was used. The simulation box was set to a size of 7 nm × 12 nm × 18.5 nm and was run at 25°C and 1 atm. The simulation was optimized and run through a series of steps: energy minimization, 100 ps NVT, 100 ps NPT, and a 50 ns production simulation.

System 3: The docked IRF4–STAT6 complex was subjected to a 50 ns molecular dynamics simulation in aqueous solution. The system was constructed using Gromacs2021 and consisted of 1 IRF4 protein molecule, 1 STAT6 protein molecule, and 222,179 water molecules, with 20 Na^+^ ions added to balance the charge in the system. The Amber99SB force field was used. The simulation box was set to a size of 13 nm × 17 nm × 18 nm and was run at 25°C and 1 atm. The simulation was optimized and run through a series of steps: energy minimization, 100 ps NVT, 100 ps NPT, followed by a 50 ns production simulation.

System 4: The docked IRF4–MyD88–IRF5 complex was subjected to a 50 ns molecular dynamics simulation in aqueous solution. The system was constructed using Gromacs2021 and consisted of 1 IRF4 protein molecule, 1 MyD88 protein molecule, 1 IRF5 protein molecule, and 260,517 water molecules, with 17 Na^+^ ions added to balance the charge in the system. The Amber99SB force field was used. The simulation box was set to a size of 25.5 nm × 26.5 nm × 25.5 nm and was run at 25°C and 1 atm. The simulation was optimized and run through a series of steps: energy minimization, 100 ps NVT, 100 ps NPT, and a 50 ns production simulation.

### Bone marrow–derived mesenchymal stem cell extraction

Bone marrow–derived mesenchymal stem cells (BM-MSCs) were isolated from the femurs and tibias of 6–8-week-old female Sprague–Dawley rats, purchased from Vital River Laboratory Animal Technology Co., Ltd. (Beijing, China; license No. SCXK (Jing) 2016-0006). Prior to bone marrow collection, animals were administered intraperitoneal pentobarbital sodium (50 mg/kg body weight; SigmaAldrich, St. Louis, Missouri, USA). The bone marrow was flushed with DMEM/F12 medium (Thermo Fisher Scientific, Waltham, MA, USA, Cat# 11320033) containing 10% fetal bovine serum (FBS; Thermo Fisher Scientific, Cat# 10099141C) and 1% penicillin/streptomycin (Gibco, #15070063). Cells were cultured at 37°C in a 5% CO₂ incubator. After 3–5 days, non-adherent cells were removed, and adherent cells were expanded for subsequent experiments. Microscopic examination revealed that the BM-MSCs exhibited a spindle-shaped or flat morphology, with oval-shaped nuclei predominantly positioned centrally or slightly eccentrically.

### Three-dimensional-printed scaffold fabrication and characterization

Scaffold construction: First, 10% GelMA hydrogel freeze-dried powder (GelMA; SunP Boyuan (Beijing) Biotechnology Co., Ltd., Beijing, China, Cat# SP-BIG01-2) was mixed with 0.25% lithium phenyl-2,4,6-trimethylbenzoylphosphinate, and 100 ng/mL IRF4 (Abcam, Cambridge, MA, USA, Cat# ab132104) was added to the bio-printing ink. The 3D printer (SUNP Biomaker, SunP (Beijing) Biotech. Co., Ltd.) was debugged to achieve a nozzle temperature of 28°C, a platform temperature of 10°C, a printing speed of 3 mm/s, incorporating ultraviolet disinfection and sterilization. The finished bio-ink was placed into the injection syringe with a needle size of 23G (internal diameter of about 350 µm). The printing process was then started. After printing, the 3D scaffold was placed at a distance of 2–3 cm from the blue light source (405 nm) and crosslinked for 15 seconds.

BM-BSCs were seeded onto scaffolds and incubated at 37°C in a 5% CO_2_ atmosphere. Cell viability was evaluated at days 7, 14, 21, and 28 using a Live/Dead Viability/Cytotoxicity Kit for mammalian cells (Cat# L3224, Thermo Fisher Scientific) according to the manufacturer’s protocol. Samples were stained with calcein-AM (live cells, green fluorescence) and ethidium homodimer-1 (dead cells, red fluorescence) for 30 minutes at room temperature (20–25°C), protected from light. Samples were visualized using confocal microscopy (LSM 880, Carl Zeiss AG, Oberkochen, Germany).

### Scanning electron microscopy

The hydrogel sample prepared above was dehydrated for 24 hours using a vacuum freeze-dryer (Alpha 1–2 LDplus, Martin Christ Gefriertrocknungsanlagen GmbH, Osterode, Germany). After freeze-drying, the samples were embedded in optimal cutting temperature compound and cryosectioned into **~**100-μm-thick slices using a cryostat microtome (Leica CM1950, Leica Microsystems GmbH, Wetzlar, Germany). The slices were placed in a vacuum chamber, uniformly coated with a thin layer of gold, and then loaded into a field emission scanning electron microscope for morphological imaging.

### Characterization of physical and chemical properties

Scaffolds were characterized using scanning electron microscopy to observe the porous architecture. Young’s modulus was measured using dynamic mechanical analysis (DMA) at 37°C. During DMA, a liquid nitrogen cooling system was used to counterbalance furnace heating and maintain the stable temperature of 37°C. The rheological properties were measured through oscillating stress scanning and frequency scanning using a rotary rheometer (MCR 302, Anton Paar GmbH, Graz, Austria) at 37°C. The contact angle was determined using a contact angle tester (OCA 15EC, DataPhysics Instruments GmbH, Filderstadt, Germany) at 37°C. Resistivity was measured using the four-probe method at 37°C. The water content of the scaffold was measured using differential scanning calorimetry and thermal gravimetric analysis (STA 449 F3 Jupiter, NETZSCH-Gerätebau GmbH, Selb, Germany) between 20 and 140°C.

### Enzyme-linked immunosorbent assay

To test the sustained release of IRF4, standard solutions of IRF4 were prepared at concentrations of 2000, 1000, 500, 250, 125, 62.5, and 31.2 pg/mL. Equal volumes (100 µL) of standard solutions and the IRF4 sample dilution (100 ng/mL) were slowly added to a 96-well plate. At various time points, 100 µL of the supernatant was collected and added back to a 96-well plate. The plate was sealed with adhesive film and incubated at 37°C for 90 minutes. After incubation, the liquid from each well was removed, and the wells were dried using absorbent paper. Then, 100 µL of anti-IRF4 antibody ELISA Kit (JL19414-48T, JONLNBIO, Shanghai, China, dilution 1:500) was added to each well, sealed, and incubated at 37°C for 60 minutes. Following incubation, 300 µL of phosphate-buffered saline (PBS; Thermo Fisher Scientific) was added to each well and allowed to soak for 1 minute. The cleaning solution was discarded, and the wells were washed three times with absorbent paper. Subsequently, 100 µL of avidin–biotin complex reagent (Vectastain Elite ABC Kit, Vector Laboratories, Burlingame, CA, USA) was added, and the plate was sealed again and incubated at 37°C for 30 minutes, followed by five washes. Finally, 90 µL of 3,3′,5,5′-tetramethylbenzidine substrate solution (Sigma-Aldrich, St. Louis, MO, USA) was added to each well, and the plate was sealed and incubated at 37°C in the dark for 30 minutes. After incubation, 100 µL of 3,3′,5,5′-tetramethylbenzidine stop solution was added to each well, and the absorbance was read at 450 nm. The absorbance values of the standard solutions were used to construct a standard curve. IRF4 concentrations at each condition were calculated from the standard curve, and the IRF4 release at each time point was statistically analyzed. IL-4 ELISA Kit (Abcam, Cat# ab100770) and IL-6 ELISA Kit (Abcam, Cat# ab234570) were used according to the manufacturer’s instructions to quantify the levels of IL-4 and IL-6 in the spinal cord. Absorbance was measured at a wavelength of 450 nm using microplate reader (Thermo Fisher Scientific, Waltham, MA, USA, Varioskan LUX). The concentrations of IL-4 and IL-6 were calculated by substituting the average optical density (OD) values of the samples into the standard curve equation.

### Scaffold degradation experiment

The 3D-printed scaffold was placed in a petri dish and continuously observed for 28 days using a stereoscope (Leica Microsystems, Wetzlar, Germany, Model: M205C).

### Animal study design and surgical procedure

**Figure 1:**
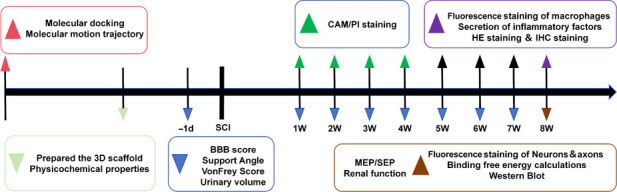
Flow chart of the animal experiments. BBB: Basso, Beattie, and Bresnahan locomotor test; CAM/PI: calcein-AM/propidium iodide; HE: hematoxylin and eosin; IHC: Immunohistochemical staining; MEP: Motor evoked potential; SCI: spinal cord injury; SEP: somatosensory evoked potential; W: week.

A total of 54 rats were included in the study. For neurophysiological assessments—including the BBB score, inclined plane support test, mechanical pain threshold test, and renal function and residual urine volume measurements—three rats were allocated to each experimental group, resulting in a total of 15 rats. Western blot analysis was conducted with three rats per group, resulting in a total of 15 rats. For macrophage M1 and M2 immunofluorescence staining, three rats were used per group, resulting in a total of 12 rats. Similarly, three rats per group were used for neuron immunofluorescence staining, cytokine secretion analysis, and immunohistochemistry, with a total of 12 rats across these experiments (**Figure 1**).

We used healthy adult female Sprague–Dawley (SD) rats, weighing 200–300 g, purchased from Vital River Laboratory Animal Technology Co., Ltd. (Beijing, China; license No. SCXK (Jing) 2016-0006). The rats were housed in a specific-pathogen-free environment with the following living conditions: a temperature of 22–24°C, humidity of 50%–60%, and a 12-hour light/dark cycle. Each rat was housed in a separate cage to ensure individual space. They were acclimated to the laboratory environment for 1 week before surgery. The rats were randomly divided into the following groups: sham (*n* = 6; T10 laminectomy removed without SCI); PBS (*n* = 12; T10 spinal cord defect model with 0.3 mL PBS injected *in situ*); 3D scaffold (*n* = 12; T10 spinal cord defect model with 3D-printed scaffold transplanted *in situ*); IRF4 (*n* = 12; T10 spinal cord defect model with IRF4 protein solution [0.3 mL, 100 ng/mL] injected *in situ*); and 3D scaffold@IRF4 (*n* = 12; T10 spinal cord defect model with the 3D-printed scaffold loaded with IRF4 [0.3 mL, 100 ng/mL] injected *in situ*).

Anesthesia was induced by intraperitoneal injection of 0.3% pentobarbital (30 mg/kg) (Sigma-Aldrich). A dorsal incision was made to expose the paraspinal muscles and tendons, which were located bilaterally along the spinal column. The hemispinal muscles, extending from the L1 region, were connected to the midline through the tendons. A marking was made at the midpoint of the intervertebral space between T10. Using gentle pressure on the abdomen to increase the intervertebral gap, the head of the rat was stabilized with the thumb and index fingers while the tail was secured. A surgical scalpel was held with the blade facing laterally, parallel to the marked intervertebral space. A precise incision was made to enter the intervertebral region, and vertical force was applied to cut through the vertebral body, with the handle tilted at a 30° angle to the uncut side. The spinal cord was completely transected, and successful SCI was confirmed by the loss of hind-limb motor function. A 2-mm segment of scaffold was implanted in the 3D scaffold and 3D scaffold@IRF4 groups. The PBS group received an injection of the same volume of PBS (0.3 mL) at the injury site and the IRF4 group was injected with IRF4 (0.3 ml, concentration of 100 ng/mL). The scaffold was securely placed between the transected spinal cord segments. After the procedure, the incision was sutured, and the rats were placed under natural light conditions to ensure they remained dry. Postoperative care included daily bladder expression and the administration of antibiotics to prevent infection.

### Behavioral assessment

Motor function recovery after SCI was assessed using multiple evaluation methods. The BBB functional score (Koopmans et al., 2005) was recorded to evaluate motor function recovery before surgery and at weekly intervals (1–8 weeks thereafter), with assessments conducted by two blinded and trained researchers. Pain sensitivity was measured using the Von Frey test (Moore et al., 2013), where different gram von Frey hairs were applied to the plantar surface of the rats, with a cut-off threshold of 20 g to prevent tissue injury. The paw withdrawal threshold was determined on the basis of consistent withdrawal responses. Gait analysis was performed using the CatWalk system (CatWalk XT 10.6) (Noldus Information Technology, Wageningen, the Netherlands), which automatically calculated gait parameters, including the regularity index, print position, and hindlimb stance, to evaluate behavioral changes after SCI.

### Neurophysiological assessments

Neurophysiological assessments were performed using standardized electrophysiological methods. Prior to the experiment, all equipment, including the stimulator (DS7A Dual Channel Stimulator, Digitimer Ltd., Welwyn Garden City, UK) and recording systems (NeuroLog System, Digitimer Ltd.), was calibrated to ensure accurate measurements. Following the surgical procedures, rats were securely positioned in a stereotaxic frame to minimize movement during data acquisition.

For motor evoked potentials (MEPs), electrical stimulation was applied to the spinal nerve, and muscle compound action potentials of the lower limbs were recorded. The integrity and synchronization of the corticospinal pathway were evaluated on the basis of the recorded signals.

Somatosensory evoked potentials (SEPs) were induced by applying sensory stimuli to the forelimbs, which generated neural responses. Detection electrodes were strategically placed along the sensory pathway to capture the evoked potentials. The latency and waveform properties of these responses were analyzed.

Residual urine measurement: Manual pressure was applied to the animal’s lower abdomen to induce bladder sphincter relaxation, facilitating spontaneous urine expulsion via the urethra. The urine was collected in a calibrated tube and its volume was recorded.

Kidney function assessment: The mouse’s tail was immersed in a thermostatically regulated water bath (Shenzhen Huayang Biotechnology Co., Ltd., Shenzhen, China, Cat# 36-0051) (45 ± 1°C) for 5 minutes. Subsequently, 3–5 mm of the distal tail was excised to permit blood collection. Following collection, the incision site was disinfected and pressure applied for hemostasis. Blood withdrawal was limited to 0.3–0.5 mL per sample. Samples were centrifuged at 800 × *g* for 20 minutes to obtain serum. Blood urea nitrogen (BUN), serum creatinine, and serum albumin were then quantified using a clinical analyzer (Hitachi Ltd., Tokyo, Japan, Hitachi 7180).

### Western blot analysis

Protein extraction was performed on spinal cord tissues, and protein concentratons were determined using a BCA assay (Thermo Fisher Scientfc). Equal amounts of protein were separated by SDS-PAGE (Bio-Rad Laboratories, Hercules, CA, USA) and transferred to polyvinylidene fluoride membranes (Merck Millipore, Burlington, MA, USA). The membranes were incubated at 4°C overnight (12–16 hours) with primary antibodies against JAK1, STAT6, IRF4, IRF5, STAT5, followed by incubation with appropriate secondary antibodies for 1 hour at room temperature (20–25°C). For experiments using Alexa Fluor 568–conjugated secondary antibodies, membranes were incubated for 1 h at room temperature protected from light. Bands were visualized using an ECL kit (Thermo Fisher Scientific) and quantified with ImageJ software (v1.52a, National Institutes of Health, Bethesda, MD, USA). Relatve protein expression was normalized to GAPDH. The primary antbodies included: rabbit ant-JAK1 (1:200, Abcam, Cat# ab138005), rabbit anti-STAT6 (1:400, Abcam, Cat# ab251555), rabbit ant-IRF4 (1:1000, Abcam, Cat# ab315394), rabbit ant-IRF5 (1:200, Abcam, Cat# ab181553), rabbit anti-STAT5 (1:1000, Abcam, Cat# ab32043), and glyceraldehyde 3-phosphate dehydrogenase (GAPDH) (1:1000, Abcam, Cat# ab9485). The secondary antbodies (all from Abcam) included: Alexa Fluor® 568 conjugated goat ant-rabbit (1:500, Cat# ab312925), HRP-conjugated goat ant-rabbit (1:5000, Cat# ab196480), HRP-conjugated goat ant-rat (1:5000, Cat# ab97051), HRP-conjugated goat ant-rat (1:20000, Cat# ab205718), Alexa Fluor 568-conjugated goat ant-rabbit (1:500, Cat# ab312939).

### Immunofluorescence staining

Spinal cord tissues were collected 8 weeks post-SCI, fixed in 4% paraformaldehyde, and cryosectioned into 10-µm slices using a cryostat (Cat# CM1860, Leica Microsystems, Wetzlar, Germany). Immunofluorescence staining was performed for CD206 (anti-inflammatory marker, rabbit, 1:100, Cat# ab64693, Abcam), CD86 (pro-inflammatory marker, rabbit, 1:200, Cat# ab53004, Abcam), β-III tubulin (pro-inflammatory marker, mouse, 1:500, Abcam, Cat# ab18207), calcein-AM (CAM) (live cell marker, 1:500, Cat# C3099, Thermo Fisher Scientific) and propidium iodide (PI) (dead cell marker, 1:1000, Cat# P3566, Thermo Fisher Scientific), and neuronal nuclei (NeuN [neural marker]; rabbit, 1:200, Abcam, Cat#ab177487, RRID: AB_2532100). Primary antibodies were incubated overnight at 4°C. After PBS washes, sections were incubated for 1 hour at room temperature (20–25°C), with the following secondary antibodies: goat anti-rabbit IgG (H+L), Alexa Fluor® 568 (1:500, Cat# ab175471, Abcam) for rabbit primaries (CD206, CD86, and NeuN) and goat anti-mouse IgG (H+L), Alexa Fluor® 488 (1:500, Cat# ab150113, Abcam) for the mouse primary (β-III tubulin). Nuclei were counterstained with DAPI (Thermo Fisher Scientific). Tissue sections were visualized using a confocal microscope (Olympus). The numbers of neurons and glia cells were calculated using ImageJ software.

### Hematoxylin and eosin and immunohistochemical staining

Eight weeks post-surgery, animals were euthanized according to ethical guidelines using a gradual-fill CO_2_ inhalation method delivered into chamber at a flow rate of 10 L/min, and spinal cord segments around the lesion site were dissected and fixed in 4% paraformaldehyde overnight at 4°C. Fixed tissues were dehydrated through a graded ethanol series, cleared with xylene, and embedded in paraffin. Sections were cut into 5–10-µm-thick slices using a microtome and mounted onto glass slides.

Hematoxylin and eosin (H&E) staining was performed to assess structural integrity. The sections were immersed in hematoxylin (Sigma-Aldrich) staining solution, stained for 5 minutes, and subsequently rinsed with tap water. The sections were further immersed in eosin (Sigma-Aldrich) staining solution, stained for 3 minutes, and washed with tap water. Finally, sections were dehydrated through graded ethanol, cleared in xylene, and mounted with a cover slip using permanent mounting medium.

For immunohistochemical staining, sections were deparaffinized in xylene, rehydrated through graded ethanol, and subjected to heat-induced epitope retrieval in citrate buffer (pH 6.0) at 95–98°C for 10–20 minutes. Endogenous peroxidase activity was blocked using 3% hydrogen peroxide (Sigma-Aldrich) for 10 minutes, followed by blocking of non-specific binding with 5%–10% normal serum at room temperature for 30 minutes. Sections were incubated overnight at 4°C with primary antibodies targeting NeuN (rabbit, 1:200, Abcam, Cat# ab177487) and glial fibrillary acidic protein (glial marker, rabbit, 1:500, Abcam, Cat# ab7260). After washing with PBS, sections were incubated with biotinylated secondary antibodies (anti-rabbit, Abcam) for 30 minutes, followed by visualization using HRP-conjugated streptavidin (Abcam) and 3,3’-diaminobenzidine (DAB) chromogen (Sigma-Aldrich) for brown staining. Counterstaining with hematoxylin (Sigma-Aldrich) was performed to visualize nuclei and the background. Finally, sections were dehydrated through graded ethanol, cleared in xylene, and mounted with coverslips using a permanent mounting medium.

### Ethics statement

The animal experiments were approved by the Animal Ethics Committee of Tianjin Medical University General Hospital on April 1, 2020, and were conducted in accordance with the National Institutes of Health Guide for the Care and Use of Laboratory Animals (8^th^ ed., National Research Council, 2011).

### Statistical analysis

No animals or data points were excluded from the analysis, which was performed by researchers blinded to the experimental conditions. Statistical analyses were performed using GraphPad Prism 8 (v8.0.2, GraphPad Software, San Diego, CA, USA, www.graphpad.com). For comparisons between multiple groups, a one-way analysis of variance (ANOVA) followed by Tukey’s *post-hoc* test was used. Results are expressed as the mean ± standard deviation (SD), and statistical significance was defined as *P*-values less than 0.05.

## Results

### Interferon regulatory factor 4 modulates macrophage polarization via competitive binding with MyD88 and interferon regulatory factor 5

**Figure 2:**
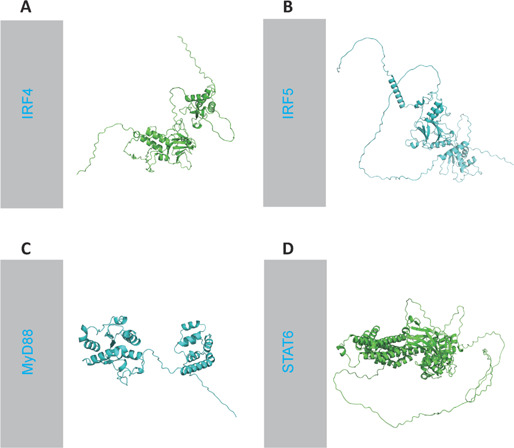
Molecular structures of the proteins IRF4 (A), IRF5 (B), MyD88 (C), and STAT6 (D). IRF4: Interferon regulatory factor 4; IRF5: Interferon regulatory factor 5; STAT6: signal transducer and activator of transcription 6.

To investigate the molecular interactions, the Uniprot database was initially used to retrieve the molecular structures of the four proteins. The full-length structures predicted by AlphaFold were then selected for further analysis (**Figure 2**).

**Figure 3:**
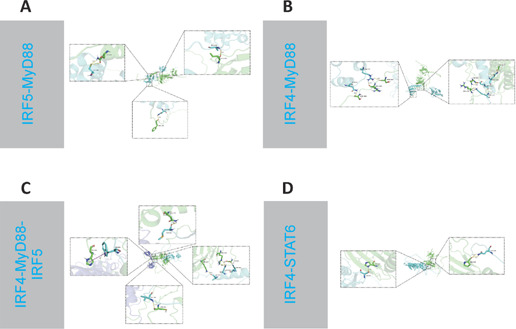
Molecular docking results for IRF5-MyD88 (A), IRF4-MyD88 (B), IRF4-MyD88-IRF5 (C), and IRF4-STAT6 (D). IRF4: Interferon regulatory factor 4; IRF5: interferon regulatory factor 5; STAT6: signal transducer and activator of transcription 6.

In the IRF5–MyD88 interaction, the core driving force of the molecular docking was the multi-residue interaction network formed by ALA-132, GEN-469, LEU-292, CS-168, and ASC-186, resulting in a total binding free energy (BFE) of –210.10 kcal/mol. Short-range hydrogen bonds and salt bridges contributed approximately 50% of the total BFE (**Figure 3A**).

For the IRF4–MyD88 interaction, the binding was primarily driven by an interaction network centered around the residues GLN-63, ARG-445, GLU-124, and LYS-127. The BFE in this case was –123.66 kcal/mol. Short-range (< 0.30 nm) polar and ionic interactions were the dominant contributors to the binding affinity, with hydrophobic residues, such as GLY-427, providing spatial complementarity (**Figure 3B**).

In the IRF4-MyD88-IRF5 complex, the driving force was the interaction network formed by ASP-283, TRP-465, LEU-471, and MET-426. The BFE between IRF4 and MyD88 was −166.59 kcal/mol, with short-range electrostatic and hydrophobic interactions accounting for approximately 60% of the binding energy. The binding energy between IRF5 and MyD88 was weaker, at −17.21 kcal/mol (**Figure 3C**).

When IRF4 and IRF5 bound to MyD88 individually, IRF5 showed a stronger binding affinity. However, in the IRF4-MyD88-IRF5 complex, electrostatic interactions between IRF4 and MyD88 weakened the binding of IRF5.

In the IRF4–STAT6 interaction, the binding was driven by a dual-core network involving HIS-233 (through coordination and hydrogen bonding) and PRO-339 (through hydrophobic stacking). The BFE in this case was −216.59 kcal/mol, with short-range interactions contributing around 50% of the total binding energy (**Figure 3D**).

The interaction between IRF5 and MyD88 played a crucial role in directing macrophage polarization toward the M1 phenotype. Molecular docking analyses indicated that IRF4 could bind to MyD88 and, in a system containing both IRF4 and IRF5, competed for the IRF5-MyD88 binding site. Additionally, IRF4 was capable of interacting with STAT6, which may have influenced the JAK/STAT signaling pathway to promote M2 macrophage polarization.

### Design and properties of three-dimensional-printed gelatin methacrylate insertable scaffold

The delivery of IRF4 protein presents notable challenges because intravenous or intraperitoneal injections lack targeting specificity, and *in situ* injections cannot maintain sustained potency (owing to cerebrospinal fluid circulation and metabolism). Therefore, a novel drug delivery system that enables the sustained release of the targeted protein is urgently needed. Our 3D-printed insertable scaffold offers a larger contact area with the spinal cord tissue than traditional scaffolds, enhancing material exchange and facilitating controlled drug release.

**Figure 4:**
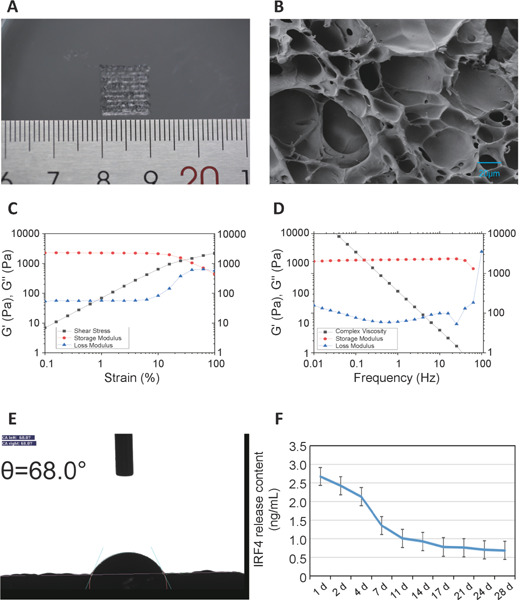
Characterization of a three-dimensional (3D)-printed insert-type scaffold. (A) Image of the 3D-printed insert-type scaffold. (B) Scanning electron microscopy image of the scaffold. (C, D) Rheological analysis demonstrating the mechanical properties of the scaffold through storage modulus, loss modulus, and complex viscosity measurements. G’: Storage modulus. G’’: Loss modulus. (E) Contact angle analysis showing the hydrophilic surface of the scaffold. (F) Drug release profile indicating the sustained release of interferon regulatory factor 4 (IRF4) over 28 days. Data in F are expressed as the mean ± SD.

The 3D-printed insertable scaffold was designed with a porous structure to promote protein adhesion (**Figure 4A** and **B**). The mechanical properties of the scaffold are primarily defined by its dominant storage modulus (G’), which remains elevated even as the frequency increases. This characteristic ensures that the scaffold maintains its structural integrity, minimizing rapid deformation and offering protection to neural tissue. Such mechanical stability is pivotal for preventing secondary damage and promoting the regeneration of neural tissue following injury (**Figure 4C** and **D**).

The contact angle (θ) of the material, which serves as an indicator of surface wettability, was less than 90°, confirming the hydrophilic nature of the scaffold’s surface (da Silva et al, 2023). This property facilitates the adhesion of key extracellular matrix proteins, such as fibronectin and laminin, which are critical for cellular attachment (Harnett et al., 2007). The hydrophilic surface mimics the polarity of the extracellular matrix, thereby enhancing cell adhesion, spreading, and proliferation, particularly for osteoblasts and stem cells. Furthermore, this surface characteristic reduces resistance to cellular contact, thereby accelerating tissue regeneration and repair. It also promotes the diffusion of water molecules, which is helpful for regulating the sustained release of IRF4 from the scaffold (**Figure 4E**).

In the drug-release experiment, IRF4 was released rapidly within the first 7 days, reaching a high concentration that coincided with the acute phase of SCI. This rapid release was advantageous for modulating the inflammatory environment during this critical period. Subsequently, the release rate declined; however, the scaffold continued to release IRF4 at a controlled rate over the subsequent 28 days. This prolonged release was essential because of the short half-life of IRF4, ensuring the maintenance of the protein’s therapeutic efficacy in the surrounding tissue and aiding in the regulation of macrophage polarization over an extended duration (**Figure 4F**).

**Figure 5:**
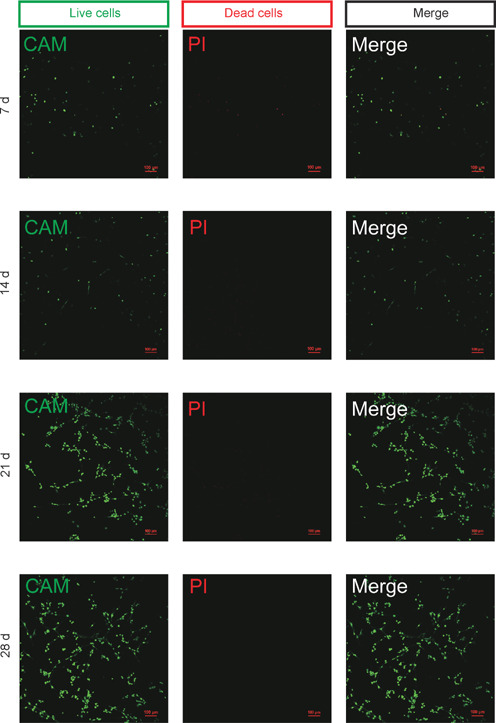
Live (green) and dead (red) staining of bone marrow–derived mesenchymal stem cells on the scaffold at 7, 14, 21, and 28 days. Scale bars: 100 µm. CAM: Calcein-AM; PI: propidium iodide.

Following a 4-week co-culture of the 3D-printed insertable scaffold with BM-MSCs, sustained cell proliferation was observed. Within the 3D hydrogel matrix, BM-MSCs formed multicellular aggregates with star-shaped or network-like architectures, establishing interactions with neighboring cells via cell-to-cell junctions. Minimal cell death was detected, further confirming high biocompatibility of the scaffold. These results suggested that the scaffold was suitable for *in situ* transplantation (**Figure 5**).

### Three-dimensional-printed scaffold enhances functional recovery following spinal cord injury

**Figure 6:**
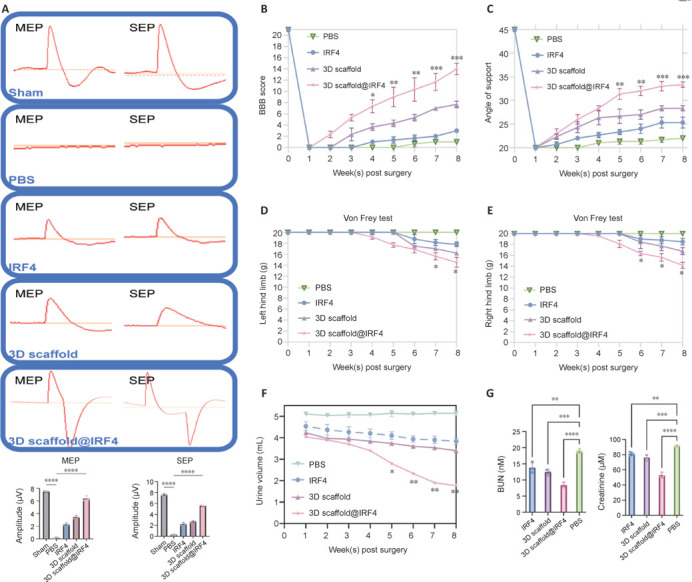
Functional recovery of rats by three-dimensional (3D)-printed scaffolds after spinal cord injury *in vivo*. (A) Representative MEP and SEP waveforms from the Sham, PBS, IRF4, 3D scaffold, and 3D scaffold@IRF4 groups. (B) BBB scores showing motor function recovery across groups over 8 weeks post-surgery. (C) Hind limb support angles measured weekly post-surgery. (D, E) Von Frey test for sensory function recovery in the left and right hind limbs, respectively. (F) Urine volume measurements over 8 weeks. (G) Blood urea nitrogen (BUN) and serum creatinine levels measured to assess kidney function. Data are expressed as the mean ± SD (*n* = 3). *P < 0.05, **P < 0.01, ***P < 0.001, ****P < 0.0001 (one-way analysis of variance followed by Tukey’s *post hoc* test). BBB: Basso, Beattie, and Bresnahan locomotor test; CAM/PI: calcein-AM/propidium iodide; IRF4: interferon regulatory factor 4; MEPs: motor-evoked potentials; PBS: phosphate-buffered saline; SEPs: somatosensory-evoked potentials.

After SCI, the waveforms and amplitudes of motor and sensory evoked potentials became almost invisible. However, animals treated with 3D-scaffold@IRF4 showed partial restoration of both amplitude and waveform morphology. Recovery was also observed in the scaffold-only and IRF4-only groups, although to a lesser extent. Minimal changes were noted in the SCI and sham control groups (**Figure 6A**).

Significant improvements in motor function recovery were observed in all treatment groups. The 3D-scaffold@IRF4 group exhibited earlier and more pronounced improvements compared with scaffold-only and IRF4-only groups. The control groups showed the least recovery (**Figure 6B** and **C**). The study examined the effect of the 3D scaffold@IRF4 group on nerve function recovery. Sensory function recovered more slowly in all groups. Early “non-recovery” in von Frey thresholds reflected frequent measurements at or above the assay cutoff, indicating right-censoring. From approximately week 3, thresholds fell below the cutoff, permitting quantifiable between-group differences. Endpoint sensory scores remained lower than motor scores across groups (**Figure 6D** and **E**).

Neurogenic bladder symptoms, such as urinary retention, were alleviated in all treated groups, with the greatest improvement observed in the 3D-scaffold@IRF4 group relative to scaffold-only, IRF4-only, and control groups (**Figure 6F**). Correspondingly, kidney function metrics were highest in the 3D-scaffold@IRF4 group, intermediate in scaffold-only and IRF4-only groups, and lowest in controls (**Figure 6G**).

### Three-dimensional-printed scaffold modulates macrophage polarization toward the M2 phenotype

**Figure 7:**
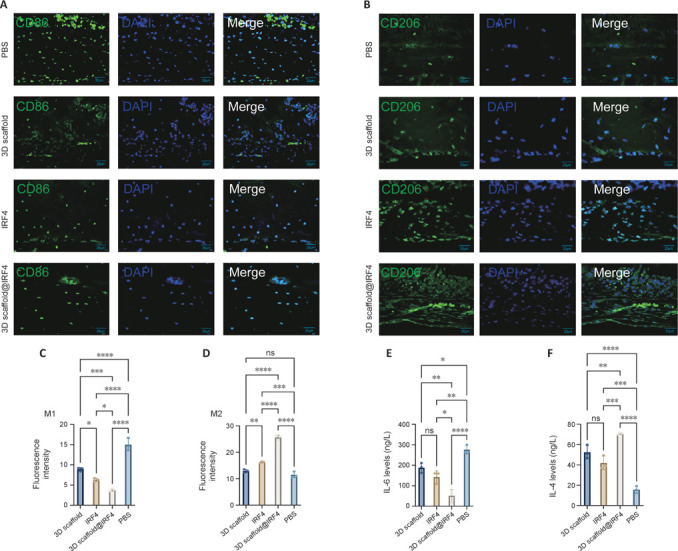
Representative immunofluorescence images and quantitative analysis of CD86 and CD206 and cytokine secretion at 8 weeks post-injury. (A) CD86 (green) and DAPI (blue) staining of spinal cord tissue 8 weeks post-injury. Scale bar: 20 μm. (B) CD206 (green) and DAPI (blue) staining of spinal cord tissue 8 weeks post-injury. Scale bar: 20 μm. (C, D) Fluorescence intensity of pro-inflammatory macrophages (M1 macrophages) and anti-inflammatory macrophages (M2 macrophages). (E) IL-6 levels (pro-inflammatory cytokines). (F) IL-4 levels (anti-inflammatory cytokine). Data are expressed as the mean ± SD (*n* = 3). *P < 0.05, **P < 0.01, ***P < 0.001, ****P < 0.0001 (one-way analysis of variance followed by Tukey’s *post hoc* test). 3D: Three-dimensional; DAPI: 4′,6-diamidino-2-phenylindole; IL: interleukin; IRF4: interferon regulatory factor 4; PBS: phosphate-buffered saline.

Eight weeks post-SCI, a substantial number of pro-inflammatory macrophages were observed in the spinal cord tissues of the PBS and 3D-scaffold groups. By contrast, the IRF4 group (with *in situ* injection) exhibited a reduced presence of pro-inflammatory macrophages, and the 3D-scaffold@IRF4 group showed a minimal number of such cells (**Figure 7A** and **C**). Similarly, very few anti-inflammatory macrophages were detected in the spinal cord of the PBS and 3D scaffold groups. The IRF4 group showed an increased number of anti-inflammatory cells, whereas the 3D-scaffold@IRF4 group demonstrated a significantly higher quantity of anti-inflammatory macrophages (**Figure 7B** and **D**).

Fluorescence-based quantitative analysis revealed notable differences in the macrophage subtypes present in the 3D-scaffold@IRF4 group compared with the other groups. Further examination of macrophage secretory function indicated no significant differences between the 3D-scaffold and IRF4 groups. However, in the 3D-scaffold@IRF4 group, there was a marked reduction in the secretion of the pro-inflammatory cytokine IL-6, coupled with a significant increase in the secretion of the anti-inflammatory cytokine IL-4. This shift in cytokine profile contributed to an inflammatory environment conducive to nerve regeneration (**Figure 7E** and **F**).

### Neural cell regeneration and tissue recovery

**Figure 8:**
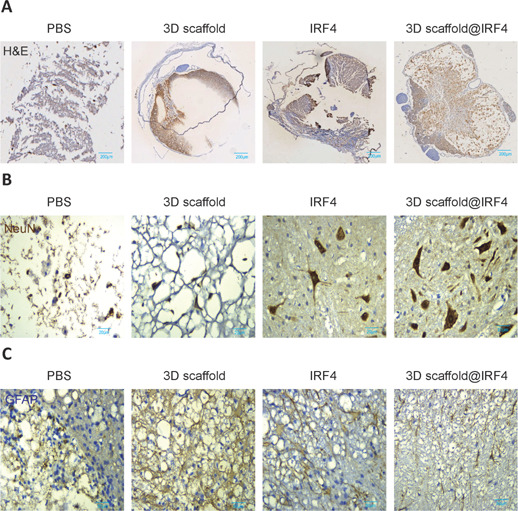
Histological and immunohistochemical analysis of spinal cord tissue. (A) Hematoxylin and eosin (H&E) staining showing the spinal cord structure in the PBS, 3D scaffold, IRF4, and 3D scaffold@IRF4 groups. (B) Immunohistochemical staining of neurons (neuronal nuclei immunoreactivity) in the spinal cord across different groups. (C) Immunohistochemical staining for astrocytes (glial fibrillary acidic protein immunoreactivity) in spinal cord tissue. 3D: Three-dimensional; IRF4: interferon regulatory factor 4; PBS: phosphate-buffered saline.

Following complete spinal cord transection, fibrous scars were observed locally, with minimal spinal cord tissue present. The 3D-scaffold group preserved the overall spinal cord structure; however, because of scaffold degradation, regenerative tissue was scarcely detectable. The IRF4 group not only failed to maintain spinal cord architecture but also exhibited fragmented regenerative tissue that was unable to effectively restore nerve function. Conversely, the 3D-scaffold@IRF4 group not only preserved the spatial structure of the spinal cord but also promoted integration with the host tissue, facilitating the growth of regenerative tissue into the scaffold (**Figure 8A**).

To further evaluate neuronal and scar tissue conditions, immunohistochemical staining was conducted. In the PBS group, neurons were almost completely absent, and a significant accumulation of astrocytes was observed. The 3D-scaffold group showed no visible neurons; however, the number of astrocytes was reduced owing to the filling effect of the scaffold. In the IRF4 group, a small number of neurons and astrocytes were detected. Notably, the 3D-scaffold@IRF4 group exhibited a large number of neurons and a near-complete absence of astrocytes, indicating enhanced neuronal regeneration and reduced glial scarring (**Figure 8B** and **C**).

**Figure 9:**
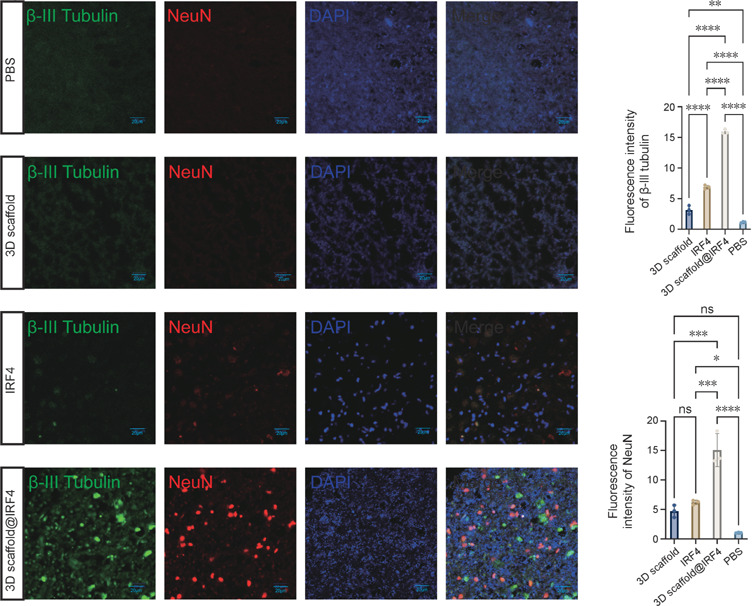
Representative immunofluorescence images of β-III tubulin (green) and NeuN (red) staining. Nuclei from each group were labeled with DAPI (blue). Data are expressed as the mean ± SD (*n* = 3). *P < 0.05, **P < 0.01, ***P < 0.001, ****P < 0.0001 (one-way analysis of variance followed by Tukey’s *post hoc* test). DAPI: 4′,6-Diamidino-2-phenylindole; NeuN: neuronal nuclei.

Further validation of nerve regeneration localized to the SCI revealed an absence of neurons and axons in the injury group and the scaffold-only group, with a very small number of neurons and regenerating axons seen in the IRF4 group. Thus, the 3D-printed scaffolds with slow-release IRF4 promoted significant regeneration of neurons and axons, with statistically significant differences between the groups (**Figure 9**).

### Molecular mechanism and experimental validation

To validate the hypothesis that IRF4 modulates macrophage polarization through competitive binding with MyD88 and IRF5, molecular dynamics simulations were conducted. These simulations were designed to test whether IRF4 competed with IRF5 for binding to MyD88, as well as whether IRF4 interacted with STAT6 to promote M2 macrophage polarization, as suggested by previous docking analyses.

**Figure 10:**
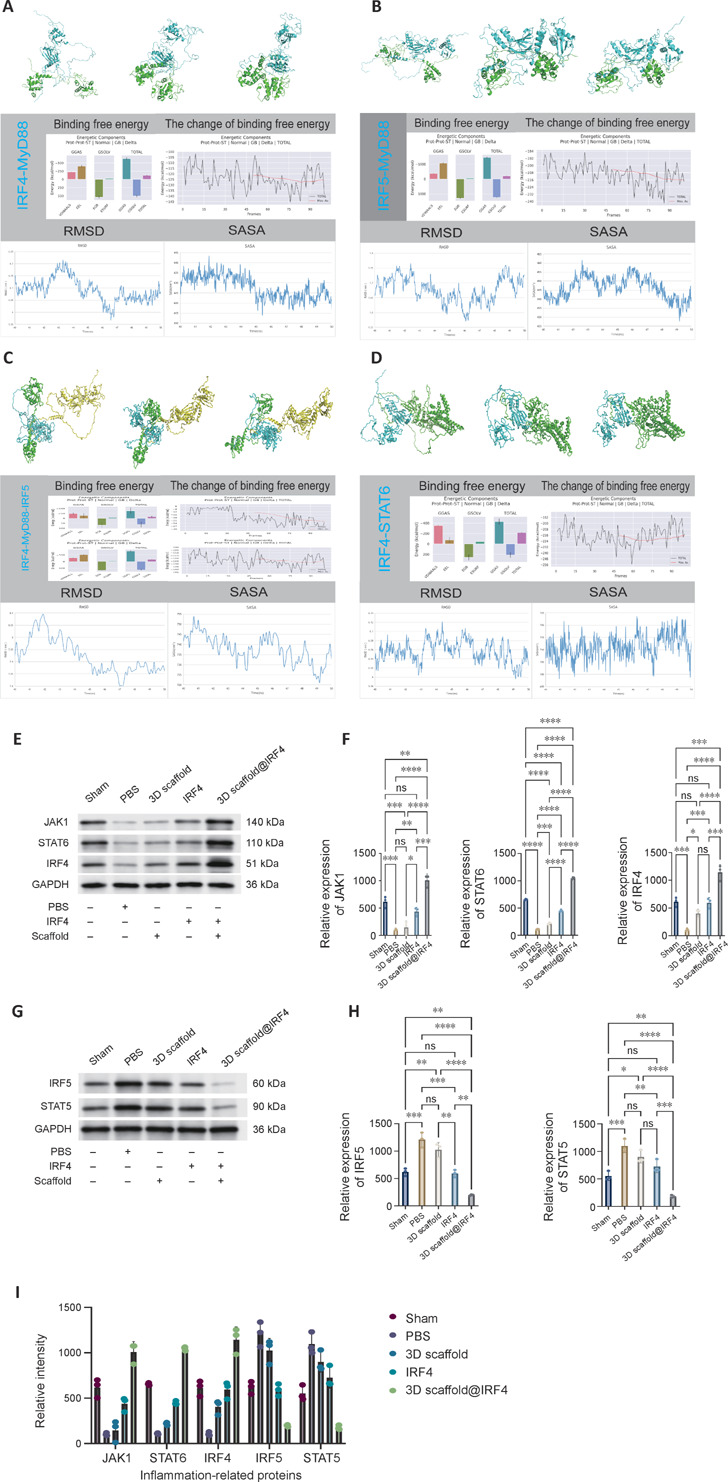
Molecular dynamics simulations and protein expression analysis. (A–D) Molecular dynamics simulation of protein‒protein interactions. (E, F) Western blot and quantification of JAK1, STAT6, and IRF4 protein expression. (G, H) Western blot and quantification of IRF5 and STAT5 protein expression. (I) Relative expression levels of JAK1, STAT6, IRF4, IRF5, and STAT5 across groups (sham, PBS, 3D scaffold, IRF4, and 3D scaffold@IRF4). Data are expressed as the mean ± SD (*n* = 3). *P < 0.05, **P < 0.01, ***P < 0.001, ****P < 0.0001 (one-way analysis of variance followed by Tukey’s *post hoc* test). GAPDH: Glyceraldehyde 3-phosphate dehydrogenase; IRF4: interferon regulatory factor 4; IRF5: interferon regulatory factor 5; JAK1: Janus kinase 1; RMSD: root mean square deviation; SASA: solvent-accessible surface area; STAT5: signal transducer and activator of transcription 5; STAT6: signal transducer and activator of transcription 6.

In the IRF4-MyD88 complex, the total BFE was −123.66 kcal/mol, with the binding energy remaining relatively stable throughout the simulation (−260 ± 5 kcal/mol), exhibiting minimal fluctuations during the initial 10 ns (ΔBFE ≤ 3 kcal/mol). This suggested that the dimeric complex possessed high binding stability. The root mean square deviation (RMSD) values fluctuated between 0.18 and 0.22 nm, indicating that the receptor–ligand interface conformation was largely conserved. The solvent-accessible surface area (SASA) showed periodic fluctuations (2.5–3.2 nm^2^), potentially because of minor conformational changes in local residues, particularly in the TIR domain of MyD88 (**Figure 10A**).

In the IRF5-MyD88 complex, the total BFE was –210.10 kcal/mol. Initially, the BFE decreased to −255 kcal/mol (ΔBFE ≈ –5 kcal/mol) before gradually increasing to −245 kcal/mol, reflecting a reduction in the binding affinity between the ligand and receptor during the simulation. The RMSD values fluctuated between 2.0 and 2.8 Å, suggesting greater flexibility at the IRF5–MyD88 interface. The SASA continuously increased (2.8–3.5 nm^2^), possibly because of exposure of the flexible side chains in IRF5 (e.g., aromatic rings) to the solvent environment (**Figure 10B**).

In the IRF4–MyD88–IRF5 ternary complex, the total BFE for IRF4–MyD88 was −166.59 kcal/mol, whereas that for IRF5–MyD88 was −17.21 kcal/mol. The BFE remained stable at −290 ± 3 kcal/mol throughout the simulation, with negligible fluctuations (ΔBFE < 2 kcal/mol), indicating that the ternary cooperative interaction significantly enhanced binding stability. The RMSD values remained consistent between 0.12 and 0.15 nm. This was markedly lower than the values observed in the dimeric systems, suggesting the formation of a rigid core through hydrophobic stacking and hydrogen bond networks. The SASA decreased to 2.0–2.4 nm^2^, possibly because of the establishment of a tight hydrophobic barrier at the ternary interface, which limited solvent molecule penetration (**Figure 10C**).

In the IRF4–STAT6 complex, the BFE was −216.59 kcal/mol. The BFE initially decreased to −270 kcal/mol before gradually increasing to −250 kcal/mol (ΔBFE ≈ +20 kcal/mol), indicating a marked reduction in binding affinity during the simulation. The RMSD values showed significant fluctuations (0.15–0.30 nm), particularly in the SH2 domain region of STAT6 (residues 400–500), where conformational shifts were evident. The SASA exhibited a bimodal distribution (2.2–2.8 nm^2^), possibly corresponding to periodic exposure or concealment of phosphorylation sites on STAT6 (**Figure 10D**).

The results confirmed that the BFE of IRF4 with MyD88 was −123.66 kcal/mol, and that IRF5 had a stronger binding affinity with MyD88 (−210.10 kcal/mol). Further analysis of the IRF4–MyD88–IRF5 system revealed that IRF4 exhibited a much higher binding affinity for MyD88 (−166.59 kcal/mol) than IRF5 (−17.21 kcal/mol), indicating that IRF4 more effectively competes for MyD88 binding. This observation supported the hypothesis that IRF4 can displace IRF5 from its binding site, favoring M2 polarization. The simulations also confirmed that electrostatic interactions and van der Waals forces played significant roles in stabilizing the IRF4-MyD88 binding, with electrostatic interactions contributing most significantly to the complex’s stability.

The binding of IRF4 to STAT6 was confirmed, with a calculated BFE of −216.59 kcal/mol. The dominant contribution to this binding came from van der Waals interactions, aligning with the hypothesis that IRF4’s interaction with STAT6 may have enhanced M2 macrophage polarization through modulation of the JAK/STAT pathway.

Protein expression analyses further corroborated these findings. In the 3D-scaffold@IRF4 group, there was a marked increase in the expression of IRF4, JAK1, and STAT6, supporting the simulation results. Conversely, the expression of IRF5 and STAT5 was significantly reduced, confirming the competitive interaction between IRF4 and IRF5 for MyD88 binding. These findings collectively validated the hypothesis that IRF4 modulated macrophage polarization through its competitive binding with MyD88 and IRF5, and its interaction with STAT6, thereby influencing the inflammatory microenvironment (**Figure 10E–I**).

In summary, IRF4 inhibited M1 macrophage polarization by competitively suppressing the binding of IRF5 to MyD88, thereby reducing the expression of pro-inflammatory proteins. Simultaneously, IRF4 promoted M2 macrophage polarization by activating the JAK1/STAT6 pathway. Through these mechanisms, IRF4 regulated inflammatory cell activity, modulated the secretion of inflammatory factors, and improved the inflammatory microenvironment, thereby facilitating nerve regeneration.

## Discussion

In this study, we found that continuous sustained-release IRF4 on 3D-printed GelMA scaffolds regulated the immune microenvironment by inhibiting M1-type macrophage polarization and promoting M2-type macrophage polarization. This reduced glial scar formation, and promoted nerve regeneration. IRF4 influences the polarization direction of macrophages by regulating the IRF/ STAT-mediated signaling pathway after SCI.

SCI is a global challenge in neural regeneration research (Zeng et al., 2023). Macrophage polarization is considered to be a primary contributor to the imbalance of the microenvironment, which subsequently hinders neural regeneration. Macrophages typically exist in two phenotypes: M1 and M2. During the acute phase of the SCI, M1 macrophages secrete pro-inflammatory cytokines, initiating an inflammatory cascade that leads to secondary damage. By contrast, M2 macrophages are generally regarded as the anti-inflammatory subtype (Kong and Gao, 2017).

IRF4 is a widely studied factor in macrophage polarization. Studies have reported that IRF4 functions through binding with MyD88 (Qin et al., 2024), subsequently activating STAT6 (Tugal et al., 2013). Additionally, IRF4 has been shown to decrease the transcription of IRF5 (Xu et al., 2011). In contrast to previous research, which has focused on protein expression levels, our work extends into ligand–receptor interactions, providing a more detailed analysis of the intermolecular interactions involved in macrophage polarization. In particular, we investigated the competitive binding of IRF4 and IRF5 to MyD88 and the role of IRF4 with STAT6, and their effects on macrophage polarization. Our molecular docking and dynamic simulation results indicated that IRF4 binds to MyD88 more effectively than IRF5 (−166.59 kcal/mol *vs.* −17.21 kcal/mol). IRF4 not only inhibits M1 macrophage polarization and suppresses the secretion of pro-inflammatory factor IL-4 by competitively interfering with IRF5–MyD88 binding, but it also synergistically binds with STAT6 to enhance the M2 macrophage polarization pathway and promotes the secretion of anti-inflammatory factor IL-6. Improving the inflammatory microenvironment after SCI is beneficial for neuronal regeneration.

IRF4 has been widely studied as a mediator of neural regeneration, particularly within the central nervous system; however, its application in SCI treatment remains underexplored. Therefore, we sought to expand our understanding of the role of IRF4 in the context of SCI. In previous research (Zhu et al., 2016), M2 macrophage transplantation reduced the IRF5/IRF4 ratio, leading to greater production of classically activated anti-inflammatory cells. Another study (Guo et al., 2014) demonstrated that neuron-specific IRF4 transgenic mice exhibited reduced infarct lesions in ischaemic stroke models, an effect that was reversed in IRF4-knockout mice. Moreover, down-regulation of IRF5 signaling by small interfering RNA or conditional knockout was shown to increase IRF4 expression, enhance M2 activation, quench proinflammatory responses, and improve stroke outcomes (Al Mamun et al., 2020). In the present study, the transplantation of IRF4 promoted functional recovery. Collectively, these findings suggest that IRF4 can shift macrophage polarization from M1 to M2, providing a potential therapeutic mechanism for modulating inflammation in neural regeneration.

Research on the application of GelMA scaffolds for the delivery of IRF4 in SCI repair is limited. Although IRF4 can modulate macrophage polarization and promote neural recovery, the delivery of the protein remains a challenge. Typically, IRF4 is delivered through intravenous, intrathecal, *in situ* injections, or via vehicle-based delivery systems. Compared with reported carrier systems, vehicle-based delivery systems have the advantage of better targeting, higher bioavailability, and reduced systemic side effects. Reported carrier systems for IRF4 delivery include retroviruses, adeno-associated viruses, and nanoparticles. For example, Guo et al. (2023) used AAVs for delivery in non-alcoholic fatty liver disease repair, but the presence of adeno-associated virus neutralizing antibodies *in vivo* limits the biological efficacy of IRF4 and reduces its therapeutic effectiveness. Xiong et al. (2023) developed a Mn_3_O_4_ nanoparticle carrier combined with IRF5-siRNA to facilitate the reversal of macrophage phenotype by downregulating IRF5 expression. However, this approach may carry potential immunogenic risks, requiring further validation to ensure its long-term safety. The GelMA scaffold is a widely used vehicle in promoting SCI repair because of its excellent biocompatibility and mechanical properties (Zhou et al., 2023). The biocompatibility of GelMA has been proven in various animal disease models, such as bone defects, brain defects, and SCI (Chen et al., 2022).

The GelMA scaffold is a modified type of hydrogel scaffold. Although synthetic polymers have been commonly used as scaffolds for SCI repair, their popularity is declining because of cytotoxicity concerns (Alexandre et al., 2014; Ferreira et al., 2018; Hashida et al., 2020). By contrast, hydrogel scaffolds have gained attention in recent years for their biocompatibility, a property that has been tested in large primates and clinical trials (Fang et al., 2021; Qin et al., 2023; Jiang et al., 2024). However, one disadvantage of natural hydrogel scaffolds is their poor mechanical stability, which is crucial for SCI models (Lan et al., 2024; Li and Gong, 2024; Yan et al., 2024). The GelMA scaffold addresses this challenge by incorporating photo-crosslinkable methacrylate groups, which provide good mechanical stability while retaining the original biocompatibility (Zhu et al., 2019; Yuan et al., 2023). GelMA-based hydrogels have been shown to support bone marrow stromal cells to form woven bone-like organs with good mechanical properties (Wang et al., 2025). In the present study, our GelMA scaffold demonstrated excellent biocompatibility and mechanical stability, as evidenced by its high storage modulus and structural integrity after 28 days. Furthermore, it ensured the sustained release of IRF4 for more than 28 days. Overall, GelMA-loaded IRF4 combined transplantation significantly improved motor, sensory, and urinary functions in SCI rats, outperforming both the IRF4-only and GelMA scaffold-only groups, thereby demonstrating a strong synergistic effect.

This study has some limitations. First, we found that sensory function recovered more slowly after SCI than motor function. This may be due to motor neurons or motor axons regenerating more effectively; however, these explanations need to be validated in future experiments. Second, the results were obtained from animal models. Therefore, it remains unclear whether these findings can be applied to human SCI treatments. Another limitation of this study is the unclear long-term effects of the IRF4-loaded scaffold, including its mechanical properties and degradation rates beyond 8 weeks. Therefore, additional interventions may be necessary for sustained recovery. Additionally, the proposed mechanisms are hypothetical and have not been validated through ligand-binding assays. Further experiments will be conducted in the future to clarify the regulatory relationship between the relevant protein molecules. Lastly, although macrophage polarization was assessed, the broader immune response, including interactions with other immune cells, was not investigated.

Future research should first focus on the long-term sustainability of the scaffold and identify potential adverse reactions. The scaffold should then be tested in primate models and clinical trials. Second, combining transcriptomics and proteomics to further explore the molecular mechanisms underlying SCI repair is recommended. This includes investigating synergistic pathways (such as mTOR and PPARγ) and transcription factors (such as TGF-β and IL-4) involved in macrophage polarization. Finally, the scaffold@IRF4 could be combined with rehabilitation techniques, such as epidural stimulation, to enhance neural plasticity, or with responsive elements (e.g., pH-sensitive, light-responsive, or sound-responsive mechanisms) to achieve precise spatiotemporal control of IRF4 release.

In conclusion, we used an interdisciplinary approach to construct a 3D-printed scaffold loaded with IRF4, overcoming the challenges of conventional delivery methods. This strategy demonstrated both the efficacy and underlying mechanisms of IRF4 in SCI repair. Our findings lay the foundation for the use of biomimetic materials in SCI treatment.

## Data Availability

*No additional data are available.*

## References

[R1] Al Mamun A, Chauhan A, Qi S, Ngwa C, Xu Y, Sharmeen R, Hazen AL, Li J, Aronowski JA, McCullough LD, Liu F (2020). Microglial IRF5-IRF4 regulatory axis regulates neuroinflammation after cerebral ischemia and impacts stroke outcomes. Proc Natl Acad Sci U S A.

[R2] Alexandre N, Ribeiro J, Gärtner A, Pereira T, Amorim I, Fragoso J, Lopes A, Fernandes J, Costa E, Santos-Silva A, Rodrigues M, Santos JD, Maurício AC, Luís AL (2014). Biocompatibility and hemocompatibility of polyvinyl alcohol hydrogel used for vascular grafting--In vitro and in vivo studies. J Biomed Mater Res A.

[R3] An N, Yang J, Wang H, Sun S, Wu H, Li L, Li M (2021). Mechanism of mesenchymal stem cells in spinal cord injury repair through macrophage polarization. Cell Biosci.

[R4] Anjum A, Yazid MD, Fauzi Daud M, Idris J, Ng AMH, Selvi Naicker A, Ismail OHR, Athi Kumar RK, Lokanathan Y (2020). Spinal cord injury: pathophysiology, multimolecular interactions, and underlying recovery mechanisms. Int J Mol Sci.

[R5] Chen Z, Wang L, Chen C, Sun J, Luo J, Cui W, Zhu C, Zhou X, Liu X, Yang H, Shi Q (2022). NSC-derived extracellular matrix-modified GelMA hydrogel fibrous scaffolds for spinal cord injury repair. NPG Asia Mater.

[R6] da Silva VA, Bobotis BC, Correia FF, Lima-Vasconcellos TH, Chiarantin GMD, De La Vega L, Lombello CB, Willerth SM, Malmonge SM, Paschon V, Kihara AH (2023). The impact of biomaterial surface properties on engineering neural tissue for spinal cord regeneration. Int J Mol Sci.

[R7] DiSabato DJ, Marion CM, Mifflin KA, Alfredo AN, Rodgers KA, Kigerl KA, Popovich PG, McTigue DM (2024). System failure: Systemic inflammation following spinal cord injury. Eur J Immunol.

[R8] Fan B, Wei Z, Feng S (2022). Progression in translational research on spinal cord injury based on microenvironment imbalance. Bone Res.

[R9] Fang G, Yang X, Wang Q, Zhang A, Tang B (2021). Hydrogels-based ophthalmic drug delivery systems for treatment of ocular diseases. Mater Sci Eng C Mater Biol Appl.

[R10] Ferreira LP, Gaspar VM, Mano JF (2018). Design of spherically structured 3D in vitro tumor models -Advances and prospects. Acta Biomater.

[R11] Fu SP, Chen SY, Pang QM, Zhang M, Wu XC, Wan X, Wan WH, Ao J, Zhang T (2022). Advances in the research of the role of macrophage/microglia polarization-mediated inflammatory response in spinal cord injury. Front Immunol.

[R12] Gao C, Li Y, Liu X, Huang J, Zhang Z (2023). 3D bioprinted conductive spinal cord biomimetic scaffolds for promoting neuronal differentiation of neural stem cells and repairing of spinal cord injury. Chem Eng J.

[R13] Gao L, Peng Y, Xu W, He P, Li T, Lu X (2020). Progress in stem cell therapy for spinal cord injury. Stem Cells Int.

[R14] Guo S, Li ZZ, Jiang DS, Lu YY, Liu Y, Gao L, Zhang SM, Lei H, Zhu LH, Zhang XD, Liu DP, Li H (2014). IRF4 is a novel mediator for neuronal survival in ischaemic stroke. Cell Death Differ.

[R15] Guo S, Feng Y, Zhu X, Zhang X, Wang H, Wang R, Zhang Q, Li Y, Ren Y, Gao X, Bian H, Liu T, Gao H, Kong X (2023). Metabolic crosstalk between skeletal muscle cells and liver through IRF4-FSTL1 in nonalcoholic steatohepatitis. Nat Commun.

[R16] Harnett EM, Alderman J, Wood T (2007). The surface energy of various biomaterials coated with adhesion molecules used in cell culture. Colloids Surf B Biointerfaces.

[R17] Hashida M (2020). Role of pharmacokinetic consideration for the development of drug delivery systems: A historical overview. Adv Drug Deliv Rev.

[R18] He W, Li ZQ, Gu HY, Pan QL, Lin FX (2024). Targeted therapy of spinal cord injury: inhibition of apoptosis is a promising therapeutic strategy. Mol Neurobiol.

[R19] He X, Li Y, Deng B, Lin A, Zhang G, Ma M, Wang Y, Yang Y, Kang X (2022). The PI3K/AKT signalling pathway in inflammation, cell death and glial scar formation after traumatic spinal cord injury: mechanisms and therapeutic opportunities. Cell Prolif.

[R20] Hellenbrand DJ, Quinn CM, Piper ZJ, Morehouse CN, Fixel JA, Hanna AS (2021). Inflammation after spinal cord injury: a review of the critical timeline of signaling cues and cellular infiltration. J Neuroinflammation.

[R21] Jiang X, Sun Y, Lyu Y, Kang H, Zhao J, Zhang K, Bian L (2024). Hydrogels for ameliorating osteoarthritis: Mechanical modulation, anti-inflammation, and regeneration. BMEMat.

[R22] Jiu J, Liu H, Li D, Li J, Liu L, Yang W, Yan L, Li S, Zhang J, Li X, Li JJ, Wang B (2024). 3D bioprinting approaches for spinal cord injury repair. Biofabrication.

[R23] Koffler J, Zhu W, Qu X, Platoshyn O, Dulin JN, Brock J, Graham L, Lu P, Sakamoto J, Marsala M, Chen S, Tuszynski MH (2019). Biomimetic 3D-printed scaffolds for spinal cord injury repair. Nat Med.

[R24] Kong X, Gao J (2017). Macrophage polarization: a key event in the secondary phase of acute spinal cord injury. J Cell Mol Med.

[R25] Koopmans GC, Deumens R, Honig WMM, Hamers FPT, Steinbusch HWM, Joosten EAJ (2005). The assessment of locomotor function in spinal cord injured rats: the importance of objective analysis of coordination. J Neurotrauma.

[R26] Lan L, Ping J, Li H, Wang C, Li G, Song J, Ying Y (2024). Skin-inspired all-natural biogel for bioadhesive interface. Adv Mater.

[R27] Li C, Xiong W, Wan B, Kong G, Wang S, Wang Y, Fan J (2022). Role of peripheral immune cells in spinal cord injury. Cell Mol Life Sci.

[R28] Li M, Wang M, Wen Y, Zhang H, Zhao GN, Gao Q (2023). Signaling pathways in macrophages: molecular mechanisms and therapeutic targets. MedComm (2020).

[R29] Li X, Gong JP (2024). Design principles for strong and tough hydrogels. Nat Rev Mater.

[R30] Liau LL, Looi QH, Chia WC, Subramaniam T, Ng MH, Law JX (2020). Treatment of spinal cord injury with mesenchymal stem cells. Cell Biosci.

[R31] Lv B, Shen N, Cheng Z, Chen Y, Ding H, Yuan J, Zhao K, Zhang Y (2022). Strategies for biomaterial-based spinal cord injury repair via the TLR4-NF-κB signaling pathway. Front Bioeng Biotechnol.

[R32] Moore SA, Hettlich BF, Waln A (2013). The use of an electronic von Frey device for evaluation of sensory threshold in neurologically normal dogs and those with acute spinal cord injury. Vet J.

[R33] National Research Council (2011). Guide for the Care and Use of Laboratory Animals.

[R34] Oraee-Yazdani S, Akhlaghpasand M, Golmohammadi M, Hafizi M, Zomorrod MS, Kabir NM, Oraee-Yazdani M, Ashrafi F, Zali A, Soleimani M (2021). Combining cell therapy with human autologous Schwann cell and bone marrow-derived mesenchymal stem cell in patients with subacute complete spinal cord injury: safety considerations and possible outcomes. Stem Cell Res Ther.

[R35] Pang QM, Chen SY, Xu QJ, Fu SP, Yang YC, Zou WH, Zhang M, Liu J, Wan WH, Peng JC, Zhang T (2021). Neuroinflammation and scarring after spinal cord injury: therapeutic roles of MSCs on inflammation and glial scar. Front Immunol.

[R36] Qin C, Qi Z, Pan S, Xia P, Kong W, Sun B, Du H, Zhang R, Zhu L, Zhou D, Yang X (2023). Advances in conductive hydrogel for spinal cord injury repair and regeneration. Int J Nanomedicine.

[R37] Qin RX, Ma XY, Han ZY, Ma SY, Shen ZJ, Lu ZH, Sun Y, Yu WL (2024). IRF2 affects LPS- and IFN-γ-induced pro-inflammatory responses, cell viability, migration and apoptosis of macrophages by regulating IRG1. J Inflamm Res.

[R38] Qiu C, Sun Y, Li J, Zhou J, Xu Y, Qiu C, Yu K, Liu J, Jiang Y, Cui W, Wang G, Liu H, Yuan W, Jiang T, Kou Y, Ge Z, He Z, Zhang S, He Y, Yu L (2023). A 3D-printed dual driving forces scaffold with self-promoted cell absorption for spinal cord injury repair. Adv Sci (Weinh).

[R39] Satoh T, Takeuchi O, Vandenbon A, Yasuda K, Tanaka Y, Kumagai Y, Miyake T, Matsushita K, Okazaki T, Saitoh T, Honma K, Matsuyama T, Yui K, Tsujimura T, Standley DM, Nakanishi K, Nakai K, Akira S (2010). The Jmjd3-Irf4 axis regulates M2 macrophage polarization and host responses against helminth infection. Nat Immunol.

[R40] Toledo B, Zhu Chen L, Paniagua-Sancho M, Marchal JA, Perán M, Giovannetti E (2024). Deciphering the performance of macrophages in tumour microenvironment: a call for precision immunotherapy. J Hematol Oncol.

[R41] Tugal D, Liao X, Jain MK (2013). Transcriptional control of macrophage polarization. Arterioscler Thromb Vasc Biol.

[R42] Visavadiya NP, Patel SP, VanRooyen JL, Sullivan PG, Rabchevsky AG (2016). Cellular and subcellular oxidative stress parameters following severe spinal cord injury. Redox Biol.

[R43] Walsh CM, Wychowaniec JK, Brougham DF, Dooley D (2022). Functional hydrogels as therapeutic tools for spinal cord injury: new perspectives on immunopharmacological interventions. Pharmacol Ther.

[R44] Walsh CM, Wychowaniec JK, Costello L, Brougham DF, Dooley D (2023). An in vitro and ex vivo analysis of the potential of GelMA hydrogels as a therapeutic platform for preclinical spinal cord injury. Adv Healthc Mater.

[R45] Wang J, Tian F, Cao L, Du R, Tong J, Ding X, Yuan Y, Wang C (2023). Macrophage polarization in spinal cord injury repair and the possible role of microRNAs: A review. Heliyon.

[R46] Wang J, Zhou D, Li R, Sheng S, Li G, Sun Y, Wang P, Mo Y, Liu H, Chen X, Geng Z, Zhang Q, Jing Y, Bai L, Xu K, Su J (2025). Protocol for engineering bone organoids from mesenchymal stem cells. Bioact Mater.

[R47] Wang Q, Wang X, Shang Z, Zhao L (2024). Mechanism and prospects of mitochondrial transplantation for spinal cord injury treatment. Stem Cell Res Ther.

[R48] Xia Y, Yang R, Wang H, Hou Y, Li Y, Zhu J, Xu F, Fu C (2022). Biomaterials delivery strategies to repair spinal cord injury by modulating macrophage phenotypes. J Tissue Eng.

[R49] Xiong T, Yang K, Zhao T, Zhao H, Gao X, You Z, Fan C, Kang X, Yang W, Zhuang Y, Chen Y, Dai J (2023). Multifunctional integrated nanozymes facilitate spinal cord regeneration by remodeling the extrinsic neural environment. Adv Sci.

[R50] Xu D, Meyer F, Ehlers E, Blasnitz L, Zhang L (2011). Interferon regulatory factor 4 (IRF-4) targets IRF-5 to regulate Epstein-Barr virus transformation. J Biol Chem.

[R51] Yan X, Huang H, Bakry AM, Wu W, Liu X, Liu F (2024). Advances in enhancing the mechanical properties of biopolymer hydrogels via multi-strategic approaches. Int J Biol Macromol.

[R52] Yeh CH, Shih HC, Hong HM, Lee SS, Yang ML, Chen CJ, Kuan YH (2015). Protective effect of wogonin on proinflammatory cytokine generation via Jak1/3-STAT1/3 pathway in lipopolysaccharide stimulated BV2 microglial cells. Toxicol Ind Health.

[R53] Yuan X, Zhu Z, Xia P, Wang Z, Zhao X, Jiang X, Wang T, Gao Q, Xu J, Shan D, Guo B, Yao Q, He Y (2023). Tough gelatin hydrogel for tissue engineering. Adv Sci (Weinh).

[R54] Yunna C, Mengru H, Lei W, Weidong C (2020). Macrophage M1/M2 polarization. Eur J Pharmacol.

[R55] Zeng CW (2023). Advancing spinal cord injury treatment through stem cell therapy: A comprehensive review of cell types, challenges, and emerging technologies in regenerative medicine. Int J Mol Sci.

[R56] Zhou B, Jiang X, Zhou X, Tan W, Luo H, Lei S, Yang Y (2023). GelMA-based bioactive hydrogel scaffolds with multiple bone defect repair functions: therapeutic strategies and recent advances. Biomater Res.

[R57] Zhu D, Yang N, Liu YY, Zheng J, Ji C, Zuo PP (2016). M2 macrophage transplantation ameliorates cognitive dysfunction in amyloid-β-treated rats through regulation of microglial polarization. J Alzheimers Dis.

[R58] Zhu M, Wang Y, Ferracci G, Zheng J, Cho NJ, Lee BH (2019). Gelatin methacryloyl and its hydrogels with an exceptional degree of controllability and batch-to-batch consistency. Sci Rep.

